# Large networks of rational agents form persistent echo chambers

**DOI:** 10.1038/s41598-018-25558-7

**Published:** 2018-08-17

**Authors:** Jens Koed Madsen, Richard M Bailey, Toby D. Pilditch

**Affiliations:** 1 School of Geography and the Environment, University of Oxford, OX1 3QY South Parks Road, Oxford, United Kingdom; 2 Department of Experimental Psychology, University College London, WC1H 0AP 26 Bedford Way, London, United Kingdom

## Abstract

Echo chambers (ECs) are enclosed epistemic circles where like-minded people communicate and reinforce pre-existing beliefs. It remains unclear if cognitive errors are necessarily required for ECs to emerge, and then how ECs are able to persist in networks with available contrary information. We show that ECs can theoretically emerge amongst error-free Bayesian agents, and that larger networks encourage rather than ameliorate EC growth. This suggests that the network structure *itself* contributes to echo chamber formation. While cognitive and social biases might exacerbate EC emergence, they are not *necessary* conditions. In line with this, we test stylized interventions to reduce EC formation, finding that system-wide *truthful* ‘educational’ broadcasts ameliorate the effect, but do not remove it entirely. Such interventions are shown to be more effective on agents newer to the network. Critically, this work serves as a formal argument for the responsibility of system architects in mitigating EC formation and retention.

## Introduction

Echo chambers (ECs) can be defined as enclosed epistemic circles where people engage with like-minded others and reinforce their shared pre-existing beliefs. They typically engender high confidence regardless of the veracity of beliefs (conspiratorial thinking (CT) is a good example of this). Recently, there has been much discussion of potential associations between ECs and political/democratic movements such as growth of populism and political polarisation^1,2^. Evidence suggests ECs have emerged and remained^3^, despite expectations that they should be increasingly untenable as social networks grow and additional, contrary information becomes available^4^.

While CT and the rise of ECs are commonly ascribed to cognitive ‘errors’, such as individual cognitive differences^5^ or fallacious reasoning^6^, some argue beliefs such as climate scepticism could emerge through rational processes^7^. The question remains open, therefore, as to whether ECs require some ‘error’, or whether ‘errors’ are merely amplifying factors of a deeper underlying process. A relevant question then is whether ECs can emerge from entirely rational social agents.

With some exceptions [e.g.^8–12^], most theoretical approaches address EC emergence using simplified analytical models where isolated agents do not interact directly^13^. However, meaningful agent interactions may generate emergent behaviour that is not observable in such models, giving rise to unexpected dynamic and adaptive group responses^14^. Strong emergence has been observed in biology^15^, social networks^16^, markets^17^, and has been used to explain behaviours such as social unrest^18^. In line with these observations, we use agent-based modelling to simulate behaviour of, and interactions between, agents^17,19–21^ equipped with reasonable cognitive assumptions. Rather than predetermining expected aggregate behavioural patterns, such as ECs, agent-based models generate these patterns naturally if (and only if) conditions allow, providing clear tests for the nature of those necessary conditions.

## Model design

We populate our model with Bayesian agents, which integrate information (according to normative, Bayesian belief revision), communicate honestly, and update their beliefs without biases (operating with perfect memory)^22,23^. We then test if many equally rational Bayesian agents, under reasonable constraints of sampling, can grow, maintain, and strengthen ECs in the context of a large social network.

While other models have explored belief diffusion^24^, cascading^25^, and opinion dynamics^26^, the current model differs in aspects concerning access to information, honesty, and speaker credibility. First, some models explore how beliefs cascade through a social system (e.g.^27,28^). By initialising the belief in one or more places in the system, some agents become the epistemic standard-bearers (an ‘agent zero’) from which beliefs can spread through the system. In comparison, no agent in the current model has privileged access to information, as all agents sample information in an identical manner at the beginning of the simulation, to form their prior belief about the world (details below). Second, in some models belief polarisation arises by manipulating levels of tolerance or credibility of agents or by allowing agents to misrepresent what they believe to be the true state of the world [e.g.^24^]. In comparison, all agents in the current model always convey what they subjectively believe is the true state of the world, and all receiving agents have complete trust in the transmitting agents. Finally, some theories of conspiratorial thinking assume cognitive differences such that conspiratorially minded agents have biases towards, for example, pattern recognition^5,6^. Agents in the current model have identical cognitive capacities.

While the above modifying factors (biases, distrust, etc.) are important in real life, we wish to explore the constrained and foundational question of whether ECs can emerge in the idealised conditions of equal, rational, honest Bayesian agents. As any observed polarisation in the model results would be neither a product of dishonesty nor a lack of access to information, this approach explores the minimal required assumptions for ECs to emergence. If ECs can emerge in such idealised conditions, it is reasonable to assume they will also emerge with less ideal agents, and perhaps more efficiently and at a faster rate. In addition, this helps in disambiguating the causes of ECs, which is useful for designing, testing, and optimising potential interventions.

In the present model, the objective true state of the world is encoded as *μ*_*true*_ = 0.5. Each agent has a belief about the value of *μ*_*true*_, described by a Gaussian distribution, with expected value *μ*_*own*_, and an associated uncertainty *σ*_*own*_. At each model time-step, agents search out other agents whose views are within a given proximity to its own; they search across a fraction of the full population, defined by the ‘search’ parameter, *α* ($\alpha \in [0,1]$Z) and accept views that fall within the range *μ*_*own*_ ± *βσ*_*own*_, where (*β* ≥ *0*). Hence, the agent’s uncertainty in its own views determine what information it will accept. As an agent receives and integrates information from other agents, it adjusts its uncertainty in its belief about *μ*_*true*_, in accordance with Bayesian updating rules (Methods). If confidence, P(H|E), increases, the agent may subsequently consider other agents, who were previously acceptable, to be extreme and no longer exchange information with them (we term this ‘pruning’). Conversely, agents widen the range of beliefs they will accept when their confidence is reduced. Choice of the pruning parameter, *β*, allows for differently ‘open-minded’ agents, between weakly confirmatory (larger *β*) to highly confirmatory (smaller *β*) (Methods).

## Simulations and Results

Simulations were conducted using a broad range of parameter values, and we show results for a representative sample: three search parameter values: α = 0.05, 0.1, 1 (recall $\alpha \in [0,1]$), and five pruning parameter values: fixed values *β* = 0.1, 0.5, 1, 2 for ‘confirmatory agents’, and values randomly assigned each time increment, *β* is ignored by ‘stochastic’ agents. In addition, two social network structures (model-R denotes random connections, which acts as a structural null hypothesis, and model-SF denotes a scale-free structure^24^, which is closer in structure to real social networks). For each parameter combination, 100 simulations were run (Methods).

**Figure 1:**
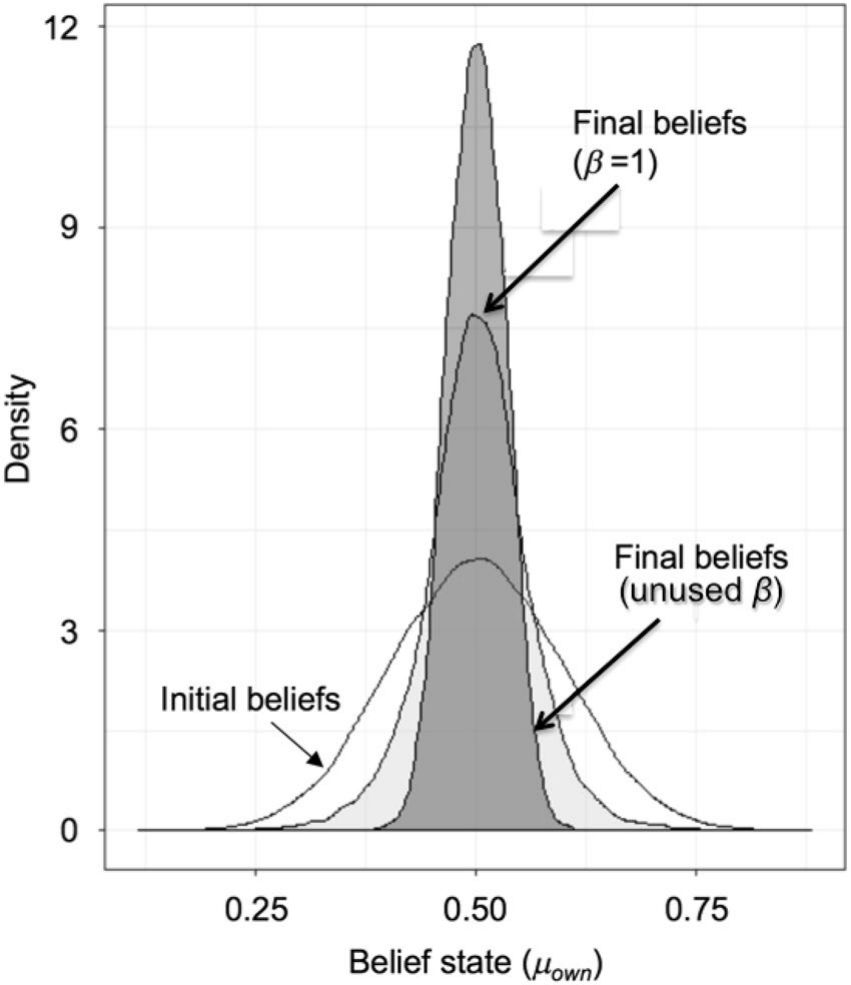
The distribution of beliefs (value of µ for each agent) shown for three cases of the random social structure model, with *α* = 1: (i) initial distribution at time zero; (ii) final beliefs (at time 200) when *β* = 1.0; (iii) final beliefs when *β* is unused (i.e. search is based solely on *α* = 1).

In a hypothetical case where all agents converge from their random initial beliefs to the objective truth (*μ*_true_ = 0.5), we would observe a progressive extinction of uncertainty for each agent over time (${\sigma }_{own}\to 0$). To test for this, at the aggregate level, we run sufficiently long simulation periods that belief distributions (frequency of *μ*_*own*_) reach a stable state (typically < 50 iterations), i.e. all agents reach subjective certainty for their *μ*_*own*_. While we do indeed observe agents’ beliefs tending towards the objective mean (Fig. 1), suggesting agents are updating their views in line with objective truth, some agents, crucially, do not end the simulation believing in the objective mean (that is, ${\mu }_{own}\ne 0.5$ for some agents). Further, in situations where individual agents can access larger numbers of other beliefs (e.g. *α* = 1), and even with relatively weak confirmatory pruning (high ‘open-mindedness’, e.g. *β* = 2), they are likely to find support for their prior beliefs regardless of how extreme these may be, leading them to become fixed. Larger networks therefore increase the numbers of agents who fail to reach the objective mean. This is made possible by the formation of ECs.

**Figure 2:**
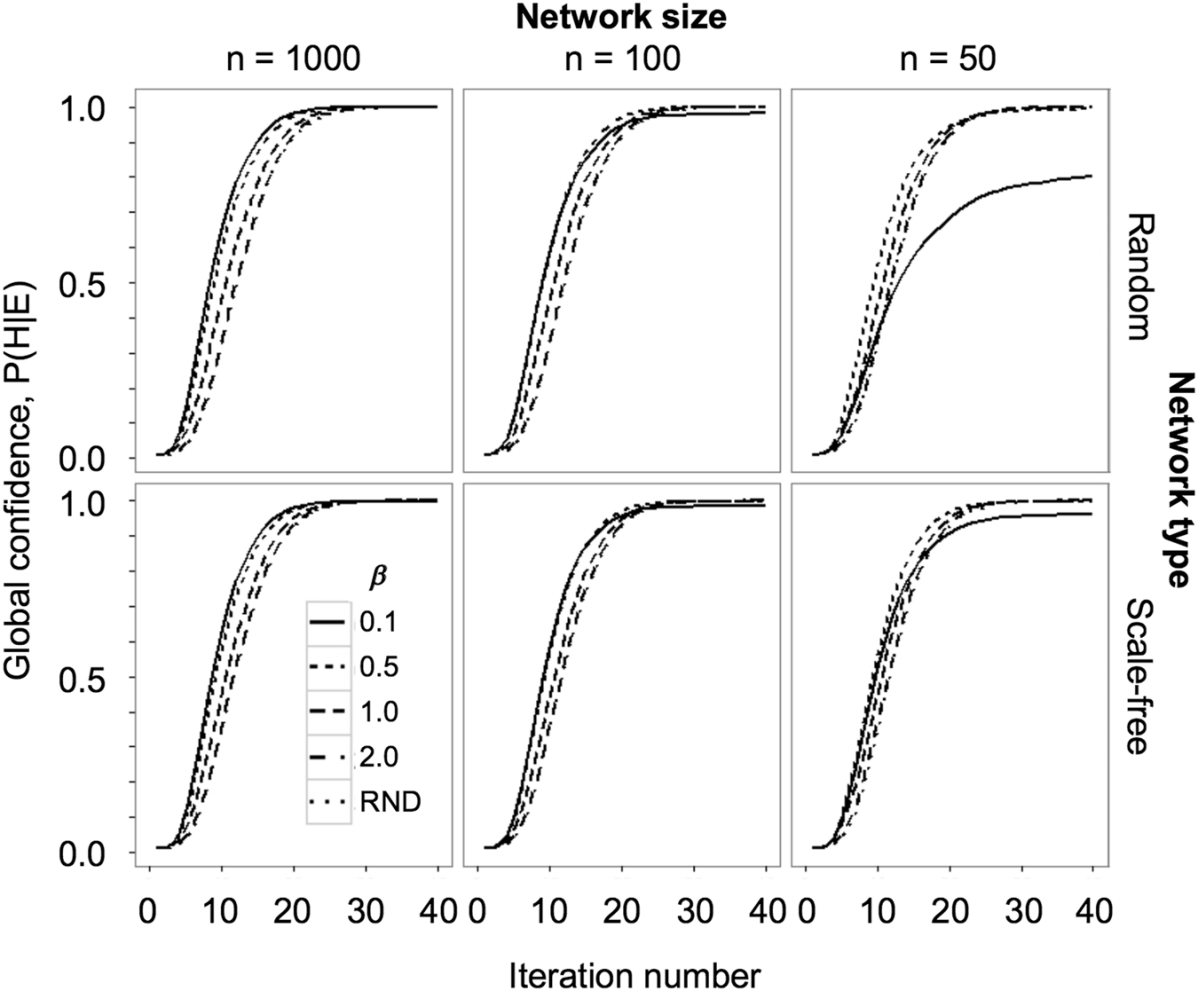
The evolution of confidence as a product of variance (*σ*_*own*_) for six simulations. Top row is stochastic placement, bottom row is Scale-Free placement (γ = 2.5). Columns depict reach in stochastic placement (*α* = 0.05, 0.1, ανδ1) and number of agents in Scale-Free placement (n = 50, 100, 1000). *β*-values range from strongly confirmatory to unused (*β* = 0.1, 0.5, 1.0, 2.0, and unused).

While agents initially have relatively low confidence in their beliefs, we observe that confidence - P(H|E), where H is the agents belief that *μ*_*own*_ = *μ*_*true*_, and E is a belief-state communicated by a member of their network, quickly increases (monotonically) at the population level as they sample information from other agents (Fig. 2a–f). Critically, this increase in confidence includes agents who entertain objectively mistaken beliefs. Although common across simulations, the final level of belief confidence achieved (at the population level) depends on the number and nature of interactions between the agents. For relatively large social networks (random and scale-free), all agents ultimately achieve high levels of confidence in their views. However, the rate at which high confidence is achieved depends (albeit weakly) on *β*, the pruning-parameter. Agents with extensive reach (large *α*, meaning a large social network) and strong confirmatory tendencies (smaller *β*) become confident relatively quickly, as they have access to ample numbers of like-minded agents. For large social networks we therefore observe faster convergence to high confidence levels with smaller *β* (Fig. 2a–b). As the size of the social network is reduced, however, *β* drives a different effect. Strongly confirmatory agents (lower *β*) in smaller social networks are often ‘starved’ of sufficient numbers of like-minded agents, and therefore their belief confidence is somewhat stymied. These observations together suggest the tendency towards greater belief confidence over time is robust, and to some extent confidence grows faster in larger social networks, although this dependence is weaker in scale-free than in random network structures.

**Figure 3:**
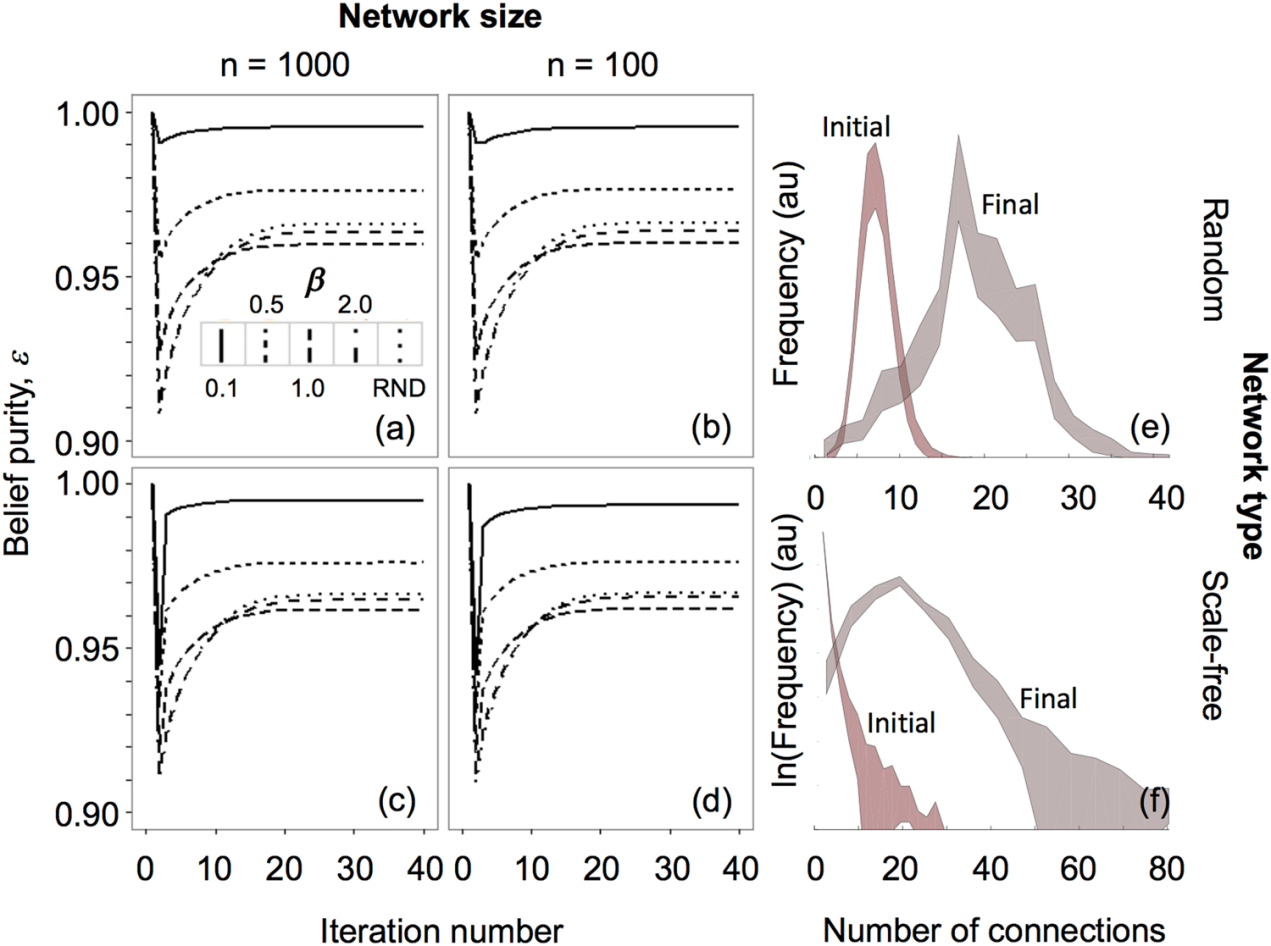
Figures a–d show the degree of epistemic similarity between nodes in a network. Top row is stochastic placement, bottom row is Scale-Free placement (γ = 2.5). Columns depict reach in stochastic placement (*α* = 0.1, and ανδ1) and number of agents in Scale-Free placement (n = 100, 1000). *β*-values range from strongly confirmatory to unused (*β* = 0.1, 0.5, 1.0, 2.0, and unused). Figures e,f show agent connection frequency distributions for 5th (‘Initial’) and 50th (‘Final’) iterations, for random and scale-free placement (95% confidence intervals over 100 repeat simulation runs).

As agents become more confident in their beliefs, and prune their existing network connections, new connections are necessarily closer to the agent’s current belief state, strengthening confidence further. There exists, therefore, an asymmetry that positively reinforces the growth of ECs: greater confidence in beliefs limits the scope for subsequently challenging those beliefs, whereas when confidence is low, a broader range of views is entertained. Consequently, once formed, the differences in beliefs *within* ECs decreases over time, while the differences *between* clusters increase. This shows the interactive and temporal nature of EC emergence and points to the importance of a systems-based approach to investigating ECs. Figure 3a–d illustrates the increase in similarity of beliefs within the network, indirectly showing the emergence of ECs through ‘belief purification’. We define belief purity $\varepsilon$, as $\varepsilon =1-({\sum }^{}{{\rm{\Delta }}}_{i}\,/{n}_{L})$, where ${{\rm{\Delta }}}_{i}\,$is the difference in *μ*_*own*_ between two linked nodes, summed over all *n*_*L*_ links in the network ($i\in [1,{n}_{L}]$). Figure 1 shows that some agents stably maintain incorrect beliefs (${\mu }_{own}\ne 0.5$), while overall there is greater ‘belief purity’ (larger $\varepsilon$), indicating the emergence of increasingly purified epistemic networks (ECs). As might be expected from earlier results (Fig. 2), stronger confirmatory pruning parameters (*β* = 0.1) produce ECs with greater conformity than weaker pruning parameters (*β* = 2). Common to all simulation results is the emergence of stable ECs with high levels of belief purism (Fig. 3a–d), populated by agents who maintain and strengthen objectively mistaken beliefs. Importantly, larger networks do not naturally ameliorate this process by providing opportunities for beliefs to be challenged.

Over the course of each simulation run, a tendency towards formation of larger social groups is observed, along with greater heterogeneity in group size across the population (Fig. 3e,f), but not the disappearance of small groups (agents with small numbers of connections). As the only mechanism to stop group formation in this model is belief disparity, these results are indicative of belief polarization.

To test the robustness of these results, we introduce a new class of anti-confirmatory agents. ‘Socratic’ agents actively seek to challenge their own views, and those of others, by sharing information only with those whose views are far from their own (but have belief distributions that overlap with their own). Introduction of these agents into the simulated social networks dilutes the tendency towards establishment of ECs and makes network purism less prevalent, through its effects on belief confidence (largely due to the attachment of Socratic agents into existing social networks). However, while purity decreased, the effect was relatively minor. That is, ECs prove difficult to disrupt, underlining the robustness of the initial findings. Indeed, when 20% of agents are Socratic, global purity is reduced only by ~2%, and only in cases where the agents are not strongly confirmatory (Socratic agents had no effect when *β* = 0.1).

**Figure 4:**
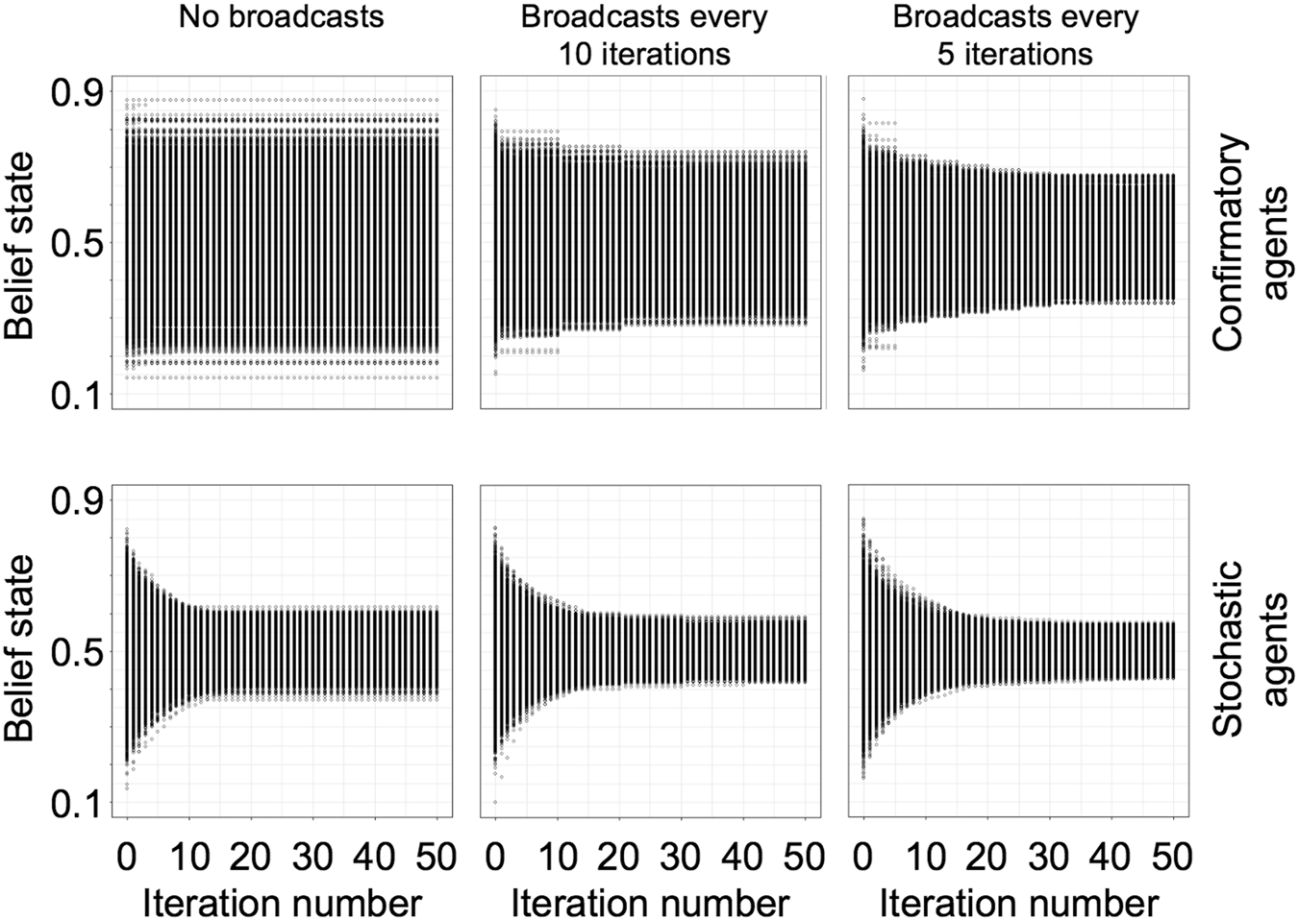
Distribution over the mean of agent beliefs over time when receiving educational broadcasting. Top row has confirmatory agents (*β*-value = 0.1), bottom row has stochastic agents with no confirmatory search parameter. Columns depict frequency of educational broadcasting (no broadcasting; every 5 ticks; every 10 ticks; every 20 ticks).

**Figure 5:**
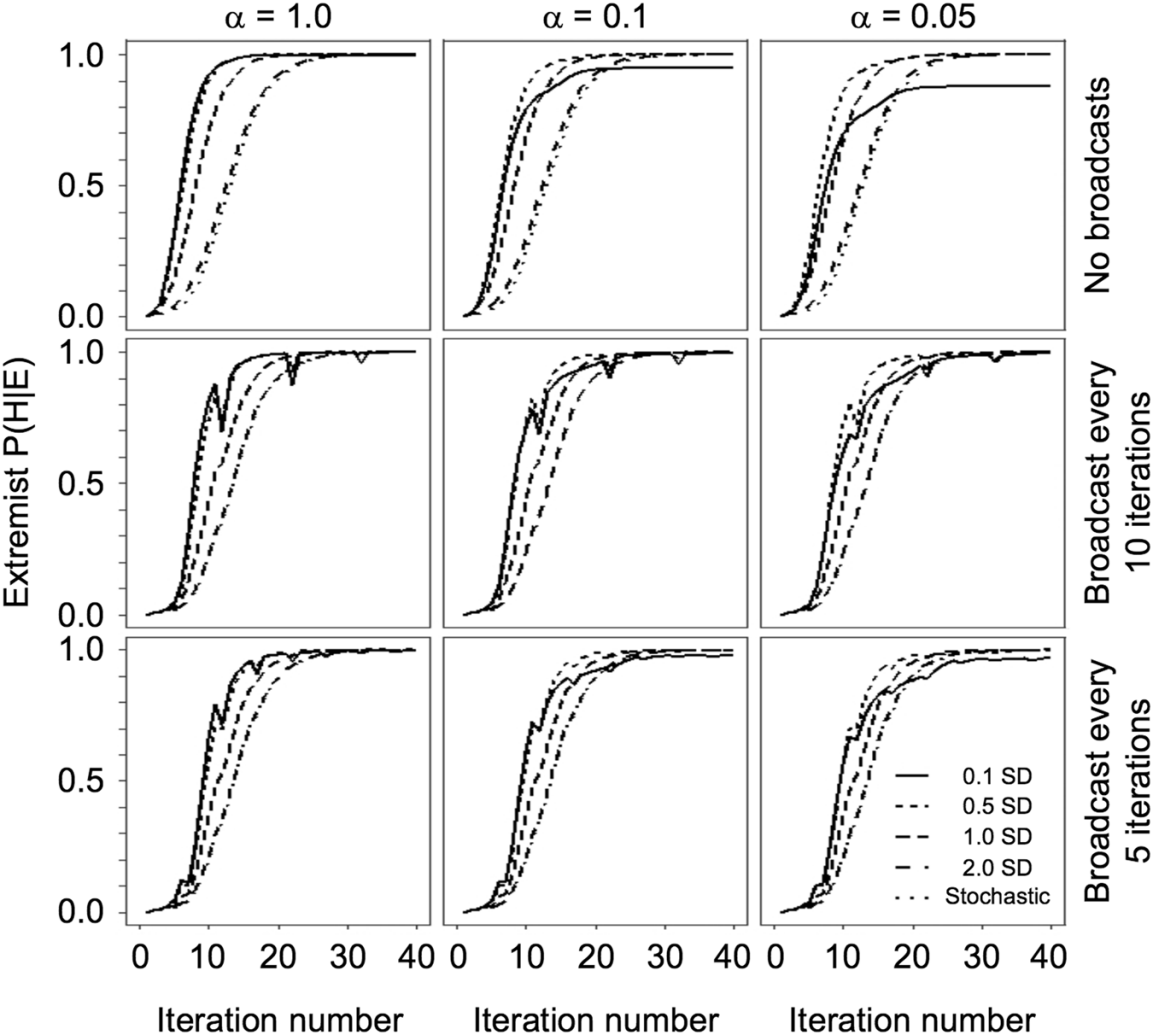
The evolution of confidence P(H|E) among extremist agents (defined as *μ*_*own*_ further than 2SD from *μ*_*true*_) when receiving educational interventions for nine simulations. Top row has no education (replication simulation), middle row broadcasts education every 10 ticks, bottom row broadcasts every 5 ticks. Columns depict reach in stochastic placement (*α* = 1, 0.1, and 0.05). *β*-values range from strongly confirmatory to unused (*β* = 0.1, 0.5, 1.0, 2.0, and unused).

As an alternative experiment, we test whether introducing an analogue of periodic educational intervention into the system could disrupt EC formation. To do this, we introduced a global broadcasting ‘signal’ with temporal frequency *v* (repeat period 1/*v*). When activated, the signal transmits the true state of the world (*μ*_true_ = 0.5) to every agent, who then revises its beliefs in accordance with Bayesian principles. To test the intervention, we employ agents with *β* = 0.1, 0.5, 1, 2, and *β* unused (Methods) (reporting only *β*-value = 0.1, as this represents the strongest test of the broadcasts), using three search parameters across the full range (α = 0.05, 0.1, 1), and four broadcast settings (no broadcasts and broadcast with *ν* = 0.2, 0.1, 0.05). The simulations show that introducing educational broadcasts decreases the amount of agents with extreme beliefs (Fig. 4). When broadcast frequency increases, this effect becomes stronger. Nonetheless, some agents continue to retain objectively inaccurate beliefs, meaning that the effect of the broadcast becomes negligible by the end of the simulation. This is observed more explicitly in Fig. 5, which shows the influence of education decreasing over the duration of the simulation (analogous to the ‘age’ of the agents). That is, the drop in individual belief confidence decreases with each intervention, as model time passes. As agents have been exposed to greater amounts of evidence over time they become more confident in their beliefs, which decreases the epistemic impact of education: early educational interventions are more effective, as entrenched mistaken views do not change even under educational interventions to the contrary. In the present case, this is due to the amount of information agents accrue from their social networks over time, rather than any change in cognitive functionality with age.

## Discussion

While it is frequently assumed that additional information leads to more accurate information integration, and that greater connectivity (e.g. via social media) is conducive to deliberative democratic debates and consensus-making, the present model points to a very different conclusion. Model results show that even in situations where agents update their beliefs in a Bayesian manner, have perfect memory, and are largely open-minded, extremist beliefs are expected to emerge and be maintained through ECs that effectively purify the local consensus. Rather than ameliorating this, larger network structures critically aggravate this process. This process appears robust to network structure and the presence of agents who actively seek to disrupt the ECs by injecting contrarian beliefs. Crucially, the model shows individual differences in cognitive functions or other components that encourage agents to become extremists are not necessary.

While the current model is an idealisation of more complex information systems, results suggests that ideals of connectivity and free speech are in potential conflict with democratic ideals concerning consensus-generation. Further, large networks are more likely to *generate* agents with extreme beliefs who do not engage with those who disagree with them. As indicated earlier, this suggests that the very networks agents inhabit contribute to EC formation regardless of the cognitive functions of the agents in the network. As such, EC formation, which may cause extremism, conspiratorial theories, and political polarisation, cannot be isolated to the individuals and their cognitive functions in question, but require models that capture interactions between agents over time. Agent-Based models simulations, as used here, are ideally placed to pursue such a challenge.

System-wide educational interventions have some utility in decreasing the number of agents with mistaken beliefs. Unsurprisingly, when education is more frequent, the effect is stronger. This suggests that at least some echo chambers *can* be broken through dissemination of information in cases where agents are willing to integrate the new information and where the educational program transmits the true state of the world. Again, both of these assumptions are idealised versions of a more complex reality. Intriguingly, the educational interventions show that education is more effective when agents are young, before they become entrenched in their beliefs.

If ECs emerge with idealised (Bayesian) reasoners, it is plausible that they will occur faster and more frequently with imperfect reasoners who have cognitive and social biases, individual differences, and flawed memory capacities. We believe these findings mirror tendencies observed during the rise of social media in recent years, where persistent extreme beliefs exist, even in the presence of ‘Socratic’ individuals who actively reach out to those with whom they disagree.

Finally, the present work makes a novel contribution by disambiguating previously conflated causes of ECs (i.e. individual-differences among agents, and the environments they inhabit). More precisely, we demonstrate that social networks are in themselves causally sufficient to promote ECs. This carries a critical implication for interventions aimed at reducing them: individual-based interventions may help reduce somewhat the harmful/erroneous thinking that promotes EC formation, but these interventions are not sufficient to remove ECs. Instead, we argue that *system-based* interventions may not only be more effective (given the potential for reducing the causal impact of *systems* on ECs), but more efficient (e.g. taking advantage of top-down system alterations) ways of reducing ECs. For instance, our (albeit simple) system-wide “educational broadcasts” illustrated the potential of altering system-architecture to reduce ECs (versus the less effective individual-based interactions of anti-confirmatory ‘Socratic’ agents). In sum, this work makes a formal argument for the responsibility of those with power over altering system architecture (and policy) in intervening on the formation of echo chambers.

## Method

To explore emergent chambers, we employ an agent-based model^17,19–21^ that allow for relevant cognitive functions (Bayesian belief revision) and agent interaction (sharing their beliefs). The model specifically explores whether Bayesian agents can become entrenched in echo chambers given repeated interactions where they share information with other agents. In the model, agents form connections with other agents via links. Through these links, the agents exchange numerical values that represent information about beliefs, updating their own beliefs in accordance with Bayes’ theorem^18,19^. In the following, we describe how the social network is structured, how they engage with other agents, and how they revise their beliefs.

Two versions of the model are used. In model-R agents are part of a randomly connected social network. In model-SF, social connections are structured as a scale-free network^29^, meaning that number of contacts follow a power law distribution (social networks frequently have such structures). To initialise the model-R network (*n*_*R*_ = 1000), agents are placed on a 100 × 100 spatial grid in random positions. For model-SF, each of *n*_*SF*_ new agent is spawned in connection with another agent with probability *p(k) = k*^*-γ*^ where *k* is the number of connections of the existing agent and γ is a constant set to 2.5 (social networks typically have 2 < γ < 3^30,31^).

Before commencing the simulation, each agent generates an initial prior belief in the hypothesis (0–1), and their degree of confidence in their estimate. To do this, each agent samples 5 values from a fixed Gaussian distribution with *µ* = 0.5, *σ* = 25. In the simulation, the objective true state is *µ* = 0.5. Thus, the distribution from which the agent samples represents noisy sampling (the *σ*) over the objective truth (the *µ*). The initial random sampling plus averaging biases the initial beliefs towards the central value of 0.5. The mean of the five sampled values provides the initial hypothesis with regard to the true value of *µ*, and confidence in this hypothesis is defined as the variance of the five values.

In model-R, parameter *α* refers to agent ‘reach’. This describes the extent of the simulated space that agents can use when forming connections with other agents (measured as Euclidean distance from each agent). For example, an agent with *α* = 0.1 is able to extend its search over a sphere with area equal to 10% of the total space. To test system sensitivity we ran simulations with *α* = 0.05, 0.1, 0.5, and 1. These values were chosen to simulate narrow range (5%) to total range (100%) and thus explore if reach influences simulation results. The observed patterns did not change drastically between 10% and 50%. Consequently, we report the 10% condition in the paper to show how quickly the patterns emerge.

For the scale-free simulations, agents share information with all others in their network. If agents prune their links (see below), they re-contact with acceptable agents in a manner that maintains the scale-free link distribution (that is, agents are more likely to form connections with well-connected agents than with poorly connected agents). To ensure common expected numbers of contacts for both model-R and model-SF, we set *n*_*SF*_ = *αn*_*R*_.

Agents update their beliefs by communicating with agents in their network (within their reach). As we are concerned with an optimal information-sharing scenario, agents transmit their *honest* epistemic state (their current estimate of *µ*) when they interact. The agent updates their belief in the hypothesis in a Bayesian manner, P(H|E) = P(H) ∗ P(E|H) / P(E), where P(H) is the agents prior belief. The likelihood ratio is calculated using two normal distribution (probability density) curves as

1 $$P(E)=\frac{1}{{\sigma }_{true}\ast \,\sqrt{2\pi }}\ast \,{e}^{-\frac{{(X-{\mu }_{true})}^{2}}{2{\sigma }_{true}^{2}}}$$

where σ_true_ refers to the *true distribution standard deviation*, *X* refers to the new piece of evidence (given by the interacting agent), and µ_true_ refers to the *true distribution mean*. This yields the probability of that evidence occurring irrespective of an agent’s particular belief-state.

2 $$P(E|H)=\frac{1}{{\sigma }_{own}\ast \,\surd (2\pi )}\ast \,{e}^{-\frac{{(X-{\mu }_{own})}^{2}}{2{\sigma }_{own}^{2}}}$$

where σ_own_ refers to *standard deviation of the agent’s belief-state distribution* and µ_own_ is the *mean of the agent’s belief-state distribution*. This yields the probability of that evidence occurring given the agent’s particular belief-state.

In this way, if the evidence is more likely given the agent’s hypothesis, the likelihood ratio is >1 and P(H|E) increases in accordance with the theorem. Conversely, if the evidence is less likely given the agent’s hypothesis, the likelihood ratio is <1 and P(H|E) decreases.

The ‘*pruning*’ parameter *β* describes the degree of belief similarity an agent requires in another before it is willing to engage with it. Agents use their own standard deviation (a measure of their confidence in the correctness of their belief) to determine whether they are willing to engage with other agents. The condition for engagement is that beliefs of other agents (${\mu }_{other}$) must fall within $({\mu }_{own}-\beta {\sigma }_{own}\,)\le {\mu }_{other}\le ({\mu }_{own}+\beta {\sigma }_{own}\,)$. The pruning parameter provides motivated information search, which is either very weakly (larger *β*) or strongly confirmatory (smaller *β*). Inclusion of pruning in the model is motivated by strong evidence that humans tend to favour (to varying degrees) media and information outlets that are in line with their ideological position (e.g., frequently reported confirmation bias^32^ and confirmatory information search strategies^33^). Additionally, pruning is motivated by practical considerations. If an agent were finite, it would be inefficient to re-sample all available information pertaining to a belief. At some point, people will consider a hypothesis settled and will refrain from seeking out new information on the issue (e.g. if a person confidently believes London is the capital of the UK, that person might not spend evenings seeking out conspiratorial theories that argue Paris is actually the capital and London is a hoax and does not exist). To simulate highly confirmatory to very open-minded agents, agents, we use a *β−*values from 0.1 to 2. The model was implemented in *NetLogo* version *5.2.1*^34^. Simulations were conducted using the RNetLogo package in R^35^.

## Electronic supplementary material

Supplementary information

## References

[1] Mann, T. E., & Ornstein, N. J. *It’s Even Worse Than It Looks: How the American Constitutional Systems Collided with the New Politics of Extremism*, Basic Books, New York, NY: USA (2012).

[2] Bakhsy E, Messing S, Adamic LA (2016). Exposure to ideologically diverse news and opinion on Facebook. Science.

[3] Flaxman, S., Goel, S. & Rao, J. M. Filter bubbles, echo chambers, and online news consumption*, Public Opinion Quarterly* 80 (special issue), 298–320 (2016).

[4] Grimes DR (2016). On the Viability of Conspiratorial Beliefs. PLoS One.

[5] Barkun, M. *A Cultural of Conspiracy: Apocalyptic Visions in Contemporary America*, University of California Press (2003).

[6] Birchall, C. *Knowledge Goes Pop: From Conspiracy Theory to Gossip*, BergPublishers, Oxford: UK (2006).

[7] Cook J, Lewandowsky S (2016). Rational irrationality: Modeling climate change belief polarization using Bayesian networks. Topics in Cognitive Sciences.

[8] Ngampruetikorn, V. & Stephens, G. J. Bias, Belief, and Consensus: Collective opinion formation on fluctuating networks, *arXiv*1512.09074v1 (2015). 10.1103/PhysRevE.94.052312 27967087

[9] Collins, P., Hahn, U., Gerber, Y. v. & Olsson, E. J. The bi-directional relationship between source characteristics and message content, in Noelle, D. C., Dale, R., Warlaumont, A. S., Yoshimi, J., Matlock, T., Jennings, C. D. & Maglio, P. P. (Eds.) (2015), *Proceedings of the 37th Annual Meeting of the Cognitive Science Society*, Austin, TX: Cognitive Science Society (2015).

[10] Olsson EJ (2011). A simulation approach to veritistic social epistemology. Episteme.

[11] Olsson, E. J. A Bayesian simulation model of group deliberation and polarization, in Zenker, F. (Ed.) *Bayesian Argumentation*, Synthese Library, Springer (2013).

[12] Vallinder A, Olsson EJ (2014). Trust and the value of overconfidence: A Bayesian perspective on social network communication. Synthese.

[13] Parunak, H. v. D., Savit, R. & Riolo, R. L. Agent-Based Modeling vs. Equation-Based Modeling: A case study and users’ guide, (pp. 10–25) *Proceedings of multi-agent systems and agent-based simulation*, Springer (1998)

[14] Ball, P. *Critical Mass: How one things leads to another*, Random House, London: UK (2005).

[15] Johnson, N. *Simply Complexity: A Clear Guide to Complexity Theory*, Oneworld Publications (2009).

[16] Schelling, T. *Micromotives and Macrobehavior*, Norton and Company, New York: NY (2006).

[17] Epstein, J. & Axtell, R. *Growing Artificial Societies*: *Social Science from the Bottom Up*, MIT Press (1996).

[18] Epstein, J. *Agent_Zero: Toward Neurocognitive foundations For Generative Social Science*, Princeton University Press (2013).

[19] Gilbert, N. *Agent-Based Models*, SAGE Publications (2008).

[20] Bandini S, Manzoni S, Vizzari G (2009). Agent Based Modeling and Simulation: An Informatics Perspective. Journal of Artificial Societies and Social Simulation.

[21] Miller, J. H. & Page, S. E. *Complex adaptive systems: An introduction to computational models of social life*, Princeton University Press (2007).

[22] Oaksford M, Chater N (1994). A Rational Analysis of the Selection Task as Optimal Data Selection. Psychological Review.

[23] Oaksford, M. & Chater, N. *Bayesian Rationality: The probabilistic approach to human reasoning*. Oxford, UK: Oxford University Press (2007). 10.1017/S0140525X09000284 19210833

[24] Duggins, P. A Psychologically-Motivated Model of Opinion Chance with Applications to American Politics, *arXiv:1406.7770* (2016).

[25] Pilditch, T. Opinion Cascades and Echo-Chambers in Online Networks: A Proof of Concept Agent-Based Model, In Gunzelmann, G., Howes, A., Tenbrink, T. & Davelaar, E. J. (Eds), *Proceedings of the 39th Annual Conference of the Cognitive Science Society*, Austin, TX: Cognitive Science Society, 943–949 (2017).

[26] Acemoglu D, Ozdaglar A (2011). Opinion dynamics and learning in social networks. Dynamic Games and Applications.

[27] Watts DJ (2002). A simple model of global cascades on random networks. Proceedings of the National Academy of Sciences of the United States of America.

[28] Watts DJ, Dodds PS (2007). Influentials, Networks, and Public Opinion Formation. Journal of Consumer Research.

[29] Amaral LAN, Scala A, Barthélémy M, Stanley HE (2000). Classes of small-world networks. PNAS.

[30] Calderelli, G. *Scale-Free Networks: Complex Webs in Nature and Technology*, Oxford University Press, Oxford: UK (2007).

[31] Clauset A, Shalizi CR, Newman MEJ (2009). Power-Law Distributions in Empirical Data. SIAM Review.

[32] Baron, J. *Thinking and deciding* (3rd ed.), New York: Cambridge University Press (2000).

[33] Klayman J, Ha Y-W (1987). Confirmation, Disconfirmation, and Information in Hypothesis Testing. Psychological Review.

[34] Wilensky, U. & Rand, W. *An Introduction to Agent-Based Modeling: Modeling Natural, Social, and Engineered Complex Systems with NetLogo*, MIT Press (2015).

[35] Thiele JC (2014). R Marries NetLogo: Introduction to the RNetLogo Package. Journal of Statistical Software.

