# Supplementary material for: Large networks of rational agents form persistent echo chambers
Source: Sci Rep. 2018 Aug 17;8:12391. doi: 10.1038/s41598-018-25558-7 (PMC6098126; doi:10.1038/s41598-018-25558-7)
Supplement: Supplementary file 1 — Supplementary information [file 41598_2018_25558_MOESM1_ESM.docx]

**Large networks of rational agents form persistent echo chambers**

Supplementary info: Appendix

Jens Koed Madsen (jens.madsen@ouce.ox.ac.uk)*

Corresponding author

School of Geography and the Environment, University of Oxford

OX1 3QY, South Parks Road, Oxford, United Kingdom

Orcid: 0000-0003-2405-8496

Richard Bailey (richard.bailey@ouce.ox.ac.uk)

School of Geography and the Environment, University of Oxford

OX1 3QY, South Parks Road, Oxford, United Kingdom

Toby D. Pilditch (t.pilditch@ucl.ac.uk)

Department of Psychology and Language Studies, University College London

WC1E 6BT, Gower Street, London, United Kingdom

**Author contributions:** Jens Koed Madsen, Richard Bailey, and Toby Pilditch have contributed equally to the conceptualisation and development of the manuscript.

**Total word count: 4505 (excluding abstract, but including all other elements)**

**Appendix A: Influence of Socratic agents on belief purism**


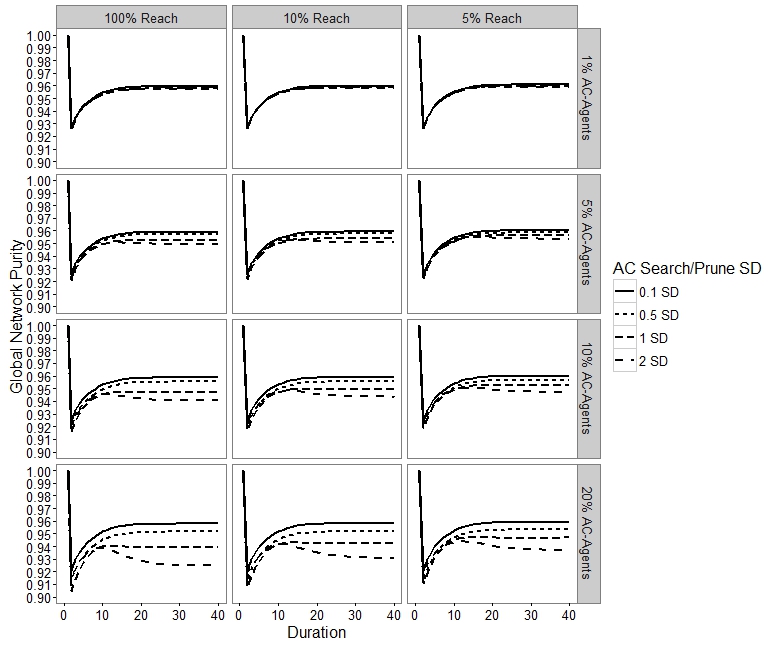


**Fig. 6 (appendix A):** Degree of epistemic similarity between nodes in a network when including Socratic agents. Horizontal columns depict reach (*α*=0.05, 0.1, ανδ1). Vertical columns depict proportion of Socratic agents relative to confirmatory agents (1%, 5%, 10%, and 20%).
